# Co-option of an extracellular protease for transcriptional control of nutrient degradation in the fungus *Aspergillus nidulans*

**DOI:** 10.1038/s42003-021-02925-1

**Published:** 2021-12-17

**Authors:** Ang Li, Chirag Parsania, Kaeling Tan, Richard B. Todd, Koon Ho Wong

**Affiliations:** 1 Faculty of Health Sciences, University of Macau, Avenida da Universidade, Taipa, Macau SAR China; 2 Gene Expression, Genomics and Bioinformatics Core, Faculty of Health Sciences, University of Macau, Avenida da Universidade, Taipa, Macau SAR China; 3 Department of Plant Pathology, Kansas State University, 1712 Claflin Road, 4024 Throckmorton Plant Sciences Center, Manhattan, KS 66506 USA; 4 Institute of Translational Medicine, Faculty of Health Sciences, University of Macau, Avenida da Universidade, Taipa, Macau SAR China; 5 MoE Frontiers Science Center for Precision Oncology, University of Macau, Avenida da Universidade, Taipa, Macau SAR China; 6 Present Address: Department of Otolaryngology-Head and Neck Surgery, First Affiliated Hospital of Guangzhou Medical University, Guangzhou, 510120 China; 7 Present Address: Gene & Stem Cell Therapy Program, Centenary Institute, Camperdown, NSW 2050 China

**Keywords:** Gene regulation, Fungal genetics, Fungal genes, Fungal biology

## Abstract

Nutrient acquisition is essential for all organisms. Fungi regulate their metabolism according to environmental nutrient availability through elaborate transcription regulatory programs. In filamentous fungi, a highly conserved GATA transcription factor AreA and its co-repressor NmrA govern expression of genes involved in extracellular breakdown, uptake, and metabolism of nitrogen nutrients. Here, we show that the *Aspergillus nidulans* PnmB protease is a moonlighting protein with extracellular and intracellular functions for nitrogen acquisition and metabolism. PnmB serves not only as a secreted protease to degrade extracellular nutrients, but also as an intracellular protease to control the turnover of the co-repressor NmrA, accelerating AreA transcriptional activation upon nitrogen starvation. PnmB expression is controlled by AreA, which activates a positive feedback regulatory loop. Hence, we uncover a regulatory mechanism in the well-established controls determining the response to nitrogen starvation, revealing functional evolution of a protease gene for transcriptional regulation and extracellular nutrient breakdown.

## Abstract (web summary)

Li et al. report the PnmB protease as a moonlighting protein in the fungus *Aspergillus nidulans* as both a secreted protease that degrades extracellular nutrients and an intracellular protease that controls the turnover of the co-repressor NmrA. These results reveal a regulatory mechanism of the drivers of the nitrogen starvation response, as well as the functional evolution of a protease gene for transcriptional regulation and extracellular nutrient breakdown.

## Introduction

Fungi have evolved remarkable metabolic versatility for utilizing diverse nutrient substrates for growth in their saprophytic, symbiotic, and pathogenic lifestyles. Evolution of fungal lifestyle correlates with their secretome, and specific classes of secreted proteases are associated with distinct environmental niches^1–4^. Secreted proteases are important in nutrient recycling via autolysis^5^ and function to promote extracellular nutrient breakdown and acquisition, a process known as lysotrophy^3,6,7^. The large repertoire of proteases expressed by fungi allows them to break down complex organic matter, usually not available to many other organisms. The extracellular release of oligopeptides and amino acids makes them available for the individual providing the extracellular protease as well as other cohabiting osmotrophic organisms, which obtain their nutrients from the environment^6,7^. This unique decomposing ability has rendered fungi an indispensable part of the ecosystem and popular partners for establishing symbiotic relationships. Moreover, secreted proteases have been implicated in the virulence of many fungal pathogens and they are employed for evasion of host recognition, colonization, and invasion, in addition to nutrient acquisition^8,9^. Therefore, proteases could offer significant advantages to fungi.

The ability to reprogram cellular metabolism according to the availability and quality of nutrients in the environment is fundamental for fungi to survive and thrive in diverse habitats^10,11^. In environments where multiple nitrogen nutrients are available, fungi first metabolize the most preferred ones, and as they become depleted, fungi switch their metabolism to utilize less preferred ones^12,13^. This preferential utilization of nutrients is a critical energy-efficient strategy for survival.

In *Aspergillus nidulans*, control of nitrogen source utilization is mediated by an elaborate regulatory mechanism in which the expression of hundreds of nitrogen metabolic genes is orchestrated by the conserved wide-domain GATA transcription factor AreA^13^. AreA modulates target gene expression according to nitrogen availability and quality. In the presence of a preferred nitrogen source, most nitrogen catabolic genes are not expressed. When the preferred nitrogen source is limiting or in the presence of an alternative nitrogen source, AreA, often together with pathway-specific transcription factors, activates the expression of specific sets of transporter and nitrogen catabolic genes for utilization of the available nutrient^13^. Moreover, in response to complete nitrogen starvation, AreA-mediated expression of certain nitrogen scavenging genes increases—a transcription response known as the nitrogen starvation response^14,15^.

Multiple control mechanisms underlie the differential regulation pattern^12,13^. First, AreA expression is controlled according to nitrogen conditions. With a preferred nitrogen source, transcription of *areA* is low, while *areA* transcripts are also unstable due to polyA tail deadenylation and CU modification^16–18^. Consequently, little AreA protein is present in the cell^19^. Transcription of *areA* is elevated through autogenous regulation under conditions when an alternative nitrogen source is available or when nitrogen nutrients are limiting^20^. At the same time, *areA* transcripts are stabilized due to lack of deadenylation and polyA tail modification^17,21^, leading to an overall increase in AreA protein levels^19^. The AreA protein is shuttled between the nucleus and cytoplasm through nuclear import and export^14,22^. During nitrogen starvation, AreA levels are further increased due to upregulation of *areA* transcription, and nuclear export of AreA is blocked, leading to high levels of AreA accumulation in the nucleus^14^.

AreA transcriptional activity also depends on control by its co-repressor NmrA. NmrA physically interacts with the AreA DNA binding domain^23,24^ and mutants with the *nmrA* gene deleted or with a C-terminal truncation of AreA also lead to partial derepression^18,25,26^. Overexpression of NmrA suppresses the nitrogen starvation response, and full suppression requires the AreA C-terminal region^19^. These previous findings highlight the importance of the levels of NmrA and its interactions with AreA for determining AreA transcriptional activation capacity.

Relatively little is known about how NmrA levels are controlled. In wild type, NmrA expression is regulated in an opposite pattern to AreA under different nitrogen conditions^19^; e.g. NmrA levels decrease with decreasing nitrogen availability and quality. The expression pattern is independent of AreA function, but is mediated by a bZIP transcription factor MeaB that binds to the *nmrA* promoter^19^. Deletion of *meaB* abolishes differential regulation of *nmrA* with only a very low basal level of NmrA present in the cell irrespective of nitrogen conditions, and as a result, *meaB*∆ mutants have a derepression phenotype similar to *nmrA*∆ mutants^19^. While MeaB is critical during favorable nitrogen conditions, transcriptional control of NmrA may be somewhat slow for reducing NmrA levels when preferred nitrogen sources become exhausted as rapid AreA-mediated transcriptional responses activating expression of nitrogen metabolism genes are required. NmrA protein levels are thought to be rapidly degraded during the transition to nitrogen starvation^27^. Three proteolytic activities against bacterial-expressed NmrA have been detected in total protein extracts of nitrogen-starved *A. nidulans* mycelia and one of them, PnmB, was purified to homogeneity based on its protease activity and cleavage of NmrA^27^. Mass spectrometry and peptide sequence analysis revealed the corresponding gene as *pnmB* (*AN2366*). The protein sequence of PnmB suggests it is a member of the trypsin-like serine protease family, which is supported by its sensitivity to the serine protease inhibitor benzamidine and its in vitro cleavage site on NmrA^27^. However, the in vivo role of *pnmB* towards NmrA degradation has not been examined.

Here, we demonstrate that PnmB degrades NmrA in vivo. PnmB expression and proteolytic activity against NmrA are dramatically induced upon nitrogen starvation and they are absolutely dependent on activation of *pnmB* transcription by AreA. Expression of PnmB causes rapid NmrA turnover and swift AreA transcriptional activation in response to nitrogen starvation. We also discover another role of PnmB as a secreted protease for extracellular protein breakdown. Finally, we uncover an interesting expansion of *pnmB*-like genes in insect-associated fungi. Overall, this work reveals a positive regulatory feedback mechanism for establishing prompt AreA activation, and also reports a protease that serves two distinct functions for the same ultimate goal of scavenging nitrogen.

## Results

### Deletion of *pnmB* has no detectable effect on growth

The PnmB protease cleaves bacterial-expressed NmrA in vitro^27^. However, the actual in vivo function in *A. nidulans* of the *pnmB* gene has not been established. We noticed a 5 bp intron containing an in-frame TAA stop codon was likely incorrectly assigned near the 3’ end of the *pnmB* annotation (Supplementary Fig. 1a). As the size of the intron is too small to be a typical intron, we reannotated the *pnmB* gene removing this proposed intron (Supplementary Fig. 1b) and confirmed our reannotation by analysis of published RNAseq data (Supplementary Fig. 1c) and introduction of a hemagglutinin (HA) epitope-tag in front of both the originally annotated and reannotated stop codons. PnmB^HA^ was only detected by western blot for the reannotated stop codon (Supplementary Fig. 1d and e). Interestingly, two distinct bands of similar mobility to the predicted molecular weight of PnmB were observed, suggesting different PnmB forms are expressed (see below). These findings demonstrate that the *pnmB* gene lacks an intron and encodes a protein of 249 amino acids (Supplementary Fig. 2).

A deletion construct to replace the entire *pnmB* coding region with the glufosinate resistance gene *Bar* from *Streptomyces hygroscopicus*^28^ was transformed into a wild-type strain (MH11036—*nkuA*∆) and glufosinate-resistant transformants were obtained. Growth of the *pnmB*∆ mutant on solid media was indistinguishable from wild type on all sole nitrogen sources analyzed (ammonium, glutamine, alanine, proline, nitrate, uric acid, acetamide, GABA, L-histidine, 2-pyrrolidinone), and on various sources of proteins (BSA, tryptone, peptone, skim milk, 25% honey) as the sole nitrogen source or sole nitrogen and carbon source (Supplementary Fig. 3a), suggesting that PnmB does not detectably affect nitrogen and carbon metabolism under the conditions tested. We also did not observe any detectable growth phenotype for the *pnmB∆* mutant under different temperature (25, 37, and 42 °C; Supplementary Fig. 3b), osmotic stress (0.4 M and 1.0 M NaCl; Supplementary Fig. 3c), and pH (pH 5.0, 6.5, and 9.0; Supplementary Fig. 3d) conditions.

### Overexpression of PnmB promotes NmrA degradation

**Fig. 1:**
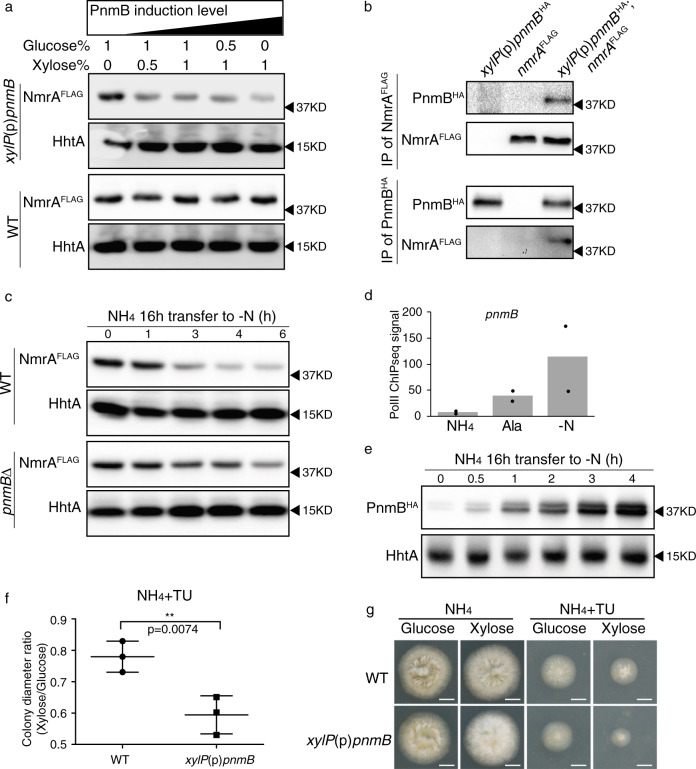
*pnmB* deletion and overexpression affects NmrA levels in vivo. **a** Western blot analysis of NmrA^FLAG^ in wild type (WT) and *xylP*(p)-*pnmB* strains grown in liquid ANM media containing glucose and xylose concentrations of 1% glucose, 1%glucose with 0.5% xylose, 1% glucose with 1% xylose, 0.5% glucose with 1% xylose, and 1% xylose. The loading order is arranged in the order of increasing PnmB induction. The histone H3 protein (HhtA) was used as a loading control. **b** Western blot analysis showing reciprocal co-IP experiments between NmrA^FLAG^ and PnmB^HA^ using anti-FLAG or anti-HA antibodies. **c** Western blot analysis of NmrA^FLAG^ in wild type (WT) and *pnmB*∆ strains in a time-course experiment upon nitrogen starvation. Strains were grown in liquid ANM media with ammonium (NH_4_) as the sole nitrogen source for 16 h (*t* = 0), then transferred to ANM nitrogen-free (-N) media for 0, 1, 3, 4, and 6 h. The histone H3 protein (HhtA) was used as a loading control. **d** A histogram showing normalized PolII ChIP-seq signal (see “Methods”) on the *pnmB* coding region under nitrogen-sufficient (NH_4_), -limiting (Ala), and -starvation (-N) conditions in wild type. Black dots present the results from two independent experiments. **e** Western blot result of PnmB^HA^ and HhtA in wild type before (*t* = 0) and after 0.5, 1, 2, 3, and 4 h of nitrogen starvation. **f** Plot showing the ratio of mean colony diameters for three replicates after 2 days growth on glucose versus xylose in the presence of 10 mM ammonium tartrate (NH_4_) and a toxic nitrogen source analog (10 mM thiourea; TU) for wild type (WT) and *xylP*(p)*pnmB* strains. Statistical significance was calculated using unpaired *t* test. Derepression is observed as inhibited colony diameter due to loss of protection against the toxic analog TU by ammonium^18,25^. **g** Colony pictures for the growth test showed in **f**. Scale bar represents 5 mm.

To test whether PnmB can degrade NmrA in vivo, we overexpressed PnmB using the strong xylose-inducible promoter of the *Penicillium chrysogenum xylP* gene^29^ under nitrogen-sufficient conditions (i.e. ammonium as the sole nitrogen source) where NmrA levels are high, and tested whether NmrA degradation could be induced. Western blot analysis showed a distinct reduction of NmrA levels when PnmB was overexpressed in a xylose-concentration-dependent manner (Fig. 1a). To rule out the possibility of non-specific degradation by the high level of PnmB protease in the cell, we measured histone H3 levels by western blot analysis (Fig. 1a) and total proteins separated by sodium dodecyl sulfate-polyacrylamide gel electrophoresis (SDS-PAGE) and stained with Coomassie Blue (Supplementary Fig. 4) as controls and observed no difference with and without PnmB overexpression. Moreover, reciprocal co-immunoprecipitation of NmrA^FLAG^ and PnmB^HA^ was observed in the presence of protease inhibitors including benzamidine known to inhibit PnmB proteolytic activity on NmrA^27^ (Fig. 1b), suggesting that the two proteins physically interact with each other. Therefore, the observed reduction in NmrA level is a specific direct effect of PnmB function.

### PnmB accelerates NmrA turnover early in nitrogen starvation

NmrA is expressed at high levels to antagonize AreA function under nitrogen sufficient conditions, but its levels decrease during nitrogen starvation thereby relieving repression of AreA for transcriptional activation^19^. To test whether PnmB is involved in degrading NmrA upon nitrogen starvation, we compared NmrA^FLAG^ protein levels of wild-type and *pnmB*∆ mutant strains during nitrogen starvation in a time course experiment. Under nitrogen sufficient conditions, NmrA^FLAG^ levels were similar between wild type and the *pnmB*∆ mutant (Fig. 1c). Consistent with previous findings, NmrA^FLAG^ levels decreased during nitrogen starvation in wild type. In contrast, NmrA^FLAG^ levels were not reduced at the same rate and extent in the *pnmB*Δ strain, indicating that NmrA turnover was slowed down in the absence of PnmB (Fig. 1c and Supplementary Fig. 5a). Therefore, this result indicates the importance of PnmB in controlling NmrA function (and therefore AreA activation) upon nitrogen starvation.

### PnmB-mediated NmrA turnover is regulated via *pnmB* expression

We next determined the expression pattern of *pnmB* under different nitrogen conditions by measuring transcription levels using chromatin immuno-precipitation followed by next-generation sequencing (ChIPseq) against the elongating form of RNA polymerase II (PolII)^30^ (Fig. 1d). Under nitrogen sufficient conditions, *pnmB* transcription levels were very low. A small increase was observed during growth on the alternative nitrogen source alanine. In stark contrast, *pnmB* transcription was induced nearly 20-fold after four hours of nitrogen starvation. Time course western blot analysis confirmed that PnmB^HA^ protein levels were barely detectable under nitrogen sufficient conditions but were substantially induced within 30 min of nitrogen starvation (Fig. 1e) and the levels continued to increase with time up to 4 h. This observation suggests that PnmB levels are the key control for NmrA turnover.

Loss of NmrA-mediated repression leads to derepression of nitrogen catabolic genes during nitrogen sufficient conditions. This derepression phenotype can be assessed in plate tests using toxic nitrogen analogs; in wild type the genes for metabolism of toxic analogs are repressed during nitrogen sufficiency and therefore normal growth occurs, whereas derepressed mutants express the genes to metabolize the analogs and show inhibited growth^25,26^. PnmB overexpression led to a derepression phenotype (i.e. inhibited growth) on solid media containing the toxic analog of urea (thiourea (TU)) in the presence of ammonium (NH_4_) (Fig. 1f, g), which is similar to the phenotype of the *nmrA*∆ mutant^25^. Taken together, the above results not only confirm the role of PnmB in NmrA turnover in vivo, but also indicate the importance of regulated PnmB expression in modulating AreA transcriptional activation and nitrogen metabolic gene expression.

### AreA activates *pnmB* to stimulate its own activation capacity

**Fig. 2:**
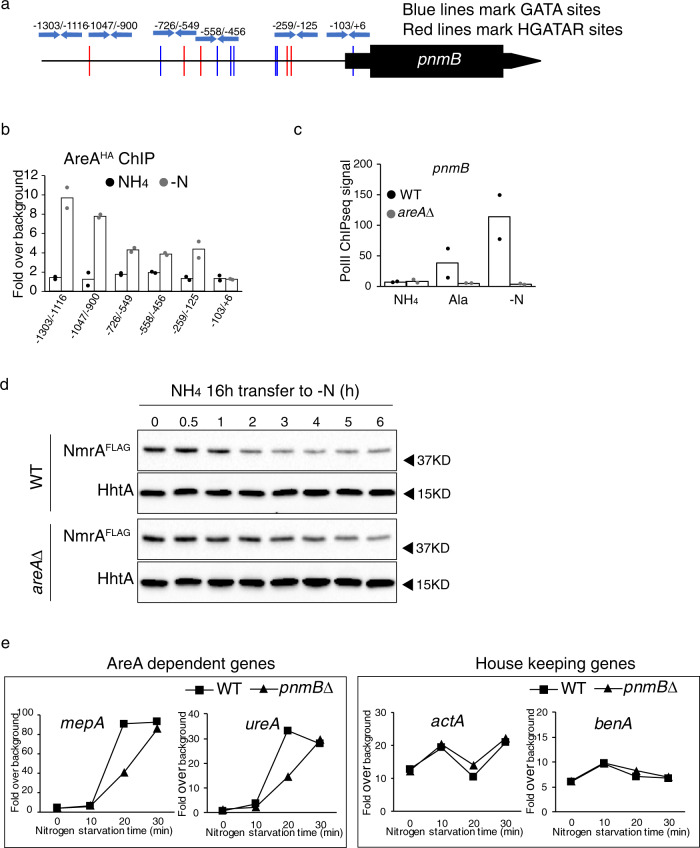
AreA directly binds to the *pnmB* promoter and activates *pnmB* expression, leading to downregulation of NmrA levels and, consequently, acceleration of the nitrogen-starvation response. **a** GATA motif distribution across the *pnmB* promoter. Locations of the previously published AreA recognition motif HGATAR (red), GATA sequences (blue), and real-time PCR primers used in ChIP analysis (blue arrows) are shown. **b** ChIP followed by real-time PCR analysis against AreA over different GATA sequences at the *pnmB* promoter using primers marked in **a** under nitrogen-sufficient (NH_4_) and -starvation (-N) conditions. Average fold over background from two independent experiments is presented (black dots for NH_4_ condition and gray dots for -N condition). **c** A histogram showing PolII ChIP-seq signal on *pnmB* coding region in WT (black dots) and *areA*∆ (gray dots) grown under nitrogen-sufficient (NH_4_), -limiting (Ala), and -starvation (-N) conditions from two independent experiments. **d** Western blot analysis of NmrA^FLAG^ in wild-type (WT) and *areA*∆ strains before and after 0.5, 1, 2, 3, 4, 5, and 6 h of nitrogen starvation. The histone H3 protein (HhtA) was used as a loading control. **e** PolII ChIP followed by real-time PCR analysis of AreA-dependent genes (*mepA* and *ureA*) and housekeeping genes upon nitrogen starvation. PolII binding is expressed as fold over background and a representative of three independent experiments are presented. See Supplementary Fig. 6 for the results of biological repeats.

AreA is the key regulator of the nitrogen starvation response^13–15,22^. The nitrogen-starvation-induced expression pattern of *pnmB* suggested a potential role of AreA in the regulation. The *pnmB* promoter sequence contains twelve GATA motifs (five belonging to the HGATAR consensus that was implicated as the AreA recognition sequence^31^) within the ~1.3 kilobase (kb) promoter region (Fig. 2a). To determine whether AreA binds to the *pnmB* promoter, ChIP followed by real-time PCR analysis (ChIP-qPCR) against AreA^HA^ was carried out using six pairs of primers spanning the *pnmB* promoter region. Consistent with minimal PnmB expression, under nitrogen sufficiency (ammonium, NH_4_), there was no detectable AreA^HA^ binding to any of these regions. In contrast, under nitrogen starvation (-N), strong AreA^HA^ ChIP signals were detected at the distal region (e.g. at ~−1300 to −900 bp), while albeit lower but significant binding signals can also be detected in the proximal region (e.g. between −726 to −125 bp) (Fig. 2b). This result showed that AreA^HA^ binds to the *pnmB* promoter at multiple locations, in response to nitrogen starvation.

We next determined whether *pnmB* transcription is dependent on AreA by comparing PolII binding at *pnmB* in wild-type and *areA*∆ mutant strains. Strikingly, both the weak (~3-fold) and strong (>150-fold) transcription induction of *pnmB* observed during growth on the alternative nitrogen source and after nitrogen starvation in wild type, respectively, was completely abolished in the *areA*∆ mutant (Fig. 2c), showing that AreA activates *pnmB* expression. This result strongly indicates a model in which AreA establishes a positive feedback loop where AreA promotes degradation of its co-repressor NmrA through activating expression of *pnmB* to stimulate its own transcriptional activation during nitrogen limitation and starvation.

Based on the model, NmrA turnover is expected to slow down in the *areA*∆ mutant during nitrogen starvation, and this is indeed the case (Fig. 2d and Supplementary Fig. 5b). Moreover, NmrA turnover kinetics in the *areA*∆ mutant is comparable to that observed in the *pnmB*∆ mutant (Fig. 1c and Supplementary Fig. 5a), consistent with the above finding that *pnmB* activation during nitrogen starvation is absolutely dependent on AreA.

We further tested the model by assessing whether PnmB-mediated NmrA turnover is important for activation of AreA-dependent gene expression immediately upon nitrogen starvation. To do this, we compared the transcription activity of AreA targets (*ureA* and *mepA*) and housekeeping control genes (*actA* and *benA*) between wild type and the *pnmB*∆ mutant in a time course of nitrogen starvation using PolII ChIP-qPCR analysis. In support of the proposed model, we found a consistent and significant delay for AreA activation in the *pnmB∆* mutant as compared to wild type (Fig. 2e and Supplementary Fig. 6). Taken together, the above results demonstrate that AreA kick-starts a regulatory feedback loop to degrade its co-repressor NmrA in order to stimulate its own activation capacity during the early phase of nitrogen starvation.

### PnmB exists as two distinct forms

**Fig. 3:**
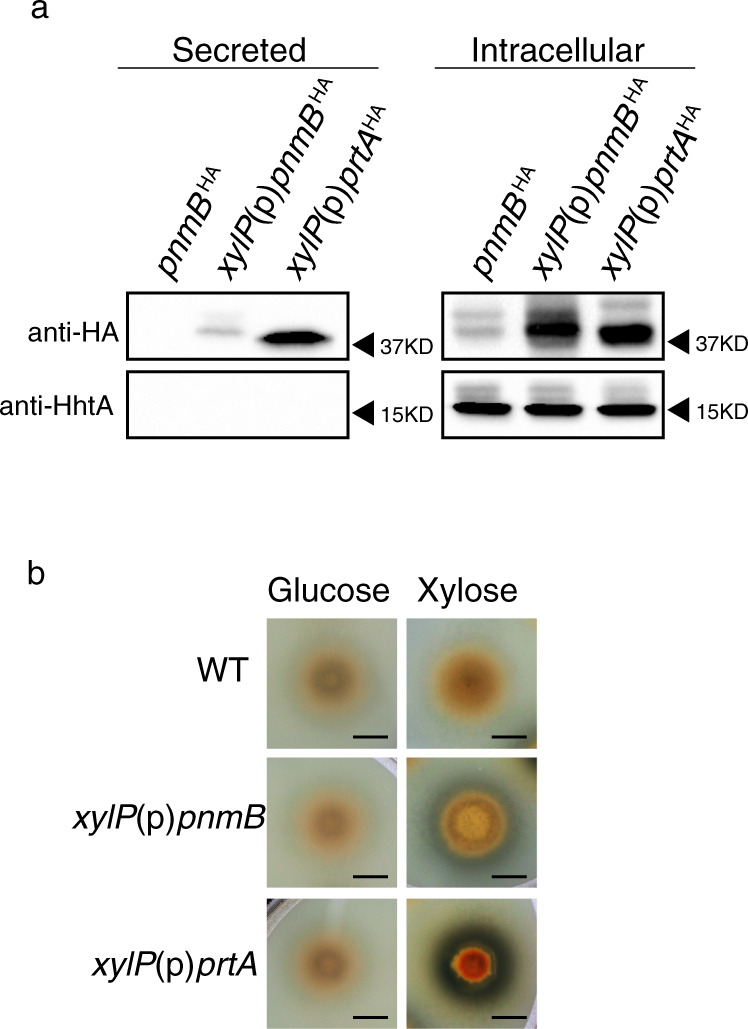
PnmB is expressed both as a secreted and an intracellular protein. **a** Western blot analysis of PnmB^HA^ and HhtA from growth media (secreted proteins) and total protein extract (intracellular proteins) of strains expressing PnmB^HA^ from its native promoter or from the strong xylose-inducible *xylP* promoter. The *xylP*(p)*prtA*^*HA*^ overexpression strain was included as a control. For the PnmB^HA^ strain, mycelia and growth media were harvested after 4 h of nitrogen starvation, while the *xylP*(p)*pnmB*^*HA*^ and *xylP*(p)*prtA*^*HA*^ strains were induced with 1% xylose for 4 h. **b** Milk clearing test of WT, *xylP*(p)*pnmB*, and *xylP*(p)*prtA*. Strains were point-inoculated to ANM + NH_4_ + 20% liquid milk plates with 1% glucose and xylose as carbon source, respectively, and incubated for 2 days. Milk clearing is observed as a halo surrounding the colony. Scale bar represents 5 mm.

The intracellular protein fraction of the wild-type PnmB^HA^ strain contains two distinct PnmB^HA^ protein bands of similar abundance in western blot analysis (Fig. 1e). The two bands were also observed in the intracellular fraction of the total protein extract when PnmB^HA^ was overexpressed (see below; Fig. 3a). There is no sign of transcript isoforms from RNAseq data. Therefore, the two PnmB forms most likely result from a post-transcriptional event. The N-terminus of PnmB carries a signal peptide (Likelihood score of 0.9939 by SignalP-5.0)^32^ that may be proteolytically cleaved, and the two forms could potentially represent the full-length and cleaved forms. Another possibility is that there could be two alternative translation initiation codons as a non-conserved methionine codon at the 18th codon position of the *pnmB* transcript may be used for internal translation initiation. At present, we cannot distinguish which of these or if other post-translational events are responsible for the two PnmB forms observed.

### PnmB is also a secreted protease

Protein sequence analysis of PnmB identified three Chymotrypsin Peptidase S1A domains and an N-terminal signal peptide, which is responsible for sorting proteins for secretion^33^, suggesting that PnmB may also be a secreted protease in addition to its intracellular role. To test this, we extracted secreted proteins from filtered growth media and assayed for the presence of PnmB. Since PnmB activity was previously found in wild type under nitrogen starvation^27^, we first performed the assay on the wild-type PnmB^HA^ strain after four hours of nitrogen starvation. A strain overexpressing PrtA^HA^, which is a known secreted protease^34^, from the strong xylose inducible *xylP* promoter^29^ was used as a positive control. While PrtA^HA^ was found in both extracts of mycelia (intracellular) and growth media (secreted) (Fig. 3a), we failed to detect PnmB^HA^ in the growth media and suspected that PnmB^HA^, even if secreted, might be present at levels too low for detection by our assay. Indeed, PnmB^HA^, when overexpressed from the *xylP* promoter, was readily detected in the growth media (Fig. 3a). The detected PnmB^HA^ and PrtA^HA^ proteins are not due to intracellular proteins from contaminating mycelia or remnants of lysed cells in the growth media, as the highly abundant histone H3 protein was not detected in the secreted fractions (Fig. 3a). It is interesting to note that only one band was found in the secreted fraction, as compared to two in the intracellular extract, and close inspection of the size of the bands showed a slight difference between secreted PnmB^HA^ and the two intracellular PnmB^HA^ forms (Supplementary Fig. 7).

Secretion of the PnmB and PrtA proteases was independently confirmed using a milk-clearing assay, in which a halo indicative of protein degradation was observed around the *xylP*(p)*pnmB*^HA^ and *xylP*(p)*prtA*^HA^ colonies when the proteins are overexpressed (Fig. 3b). Therefore, PnmB can be secreted and functions as an extracellular protease, serving two distinct but related functions—direct nutrient breakdown and global nitrogen regulation of genes for nutrient acquisition.

### Expansions of *pnmB*-like genes in many entomo-fungal species

**Fig. 4:**
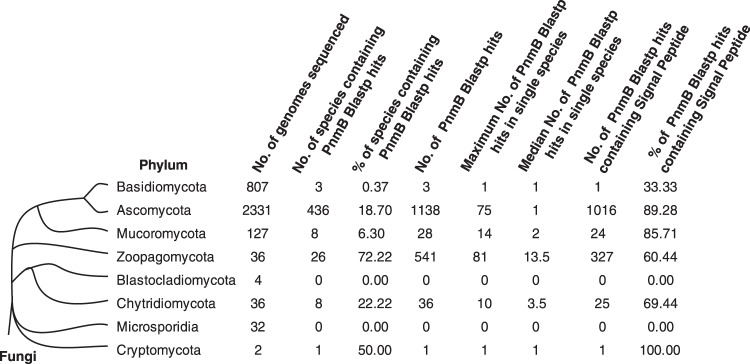
Distribution of PnmB BLASTP hits in the fungal kingdom. Phylum-level distribution of PnmB BLASTP hits across eight phyla of the fungal kingdom.

To study the evolutionary conservation of *pnmB* in fungi, we performed BLASTP searches of PnmB against the 3375 publicly available fungal genomes in NCBI and JGI databases, which represent major fungal lineages. PnmB BLASTP hits were not evenly distributed across fungal lineages (Fig. 4 and Supplementary Data 1). Notably, fungal species in the *Zoopagomycota* phylum generally carry multiple *pnmB-*like genes (Fig. 4 and Supplementary Data 2). Interestingly, those species carrying the highest number of *pnmB* homologs are associated with insects or arthropods as a pathogen, commensal or symbiont (Table 1). Therefore, the *pnmB* gene family has expanded in many entomo-fungi.

**Table 1:** Expansion of PnmB in insect-associated fungi.

Fungal species (Phylum)^a^	No. of PnmB BlastP hits	Entomo or Arthropod related	Reference
*Entomophthora muscae* (Zoopagomycota)	81	Yes	^47^
*Metarhizium anisopliae* (Ascomycota)	75	Yes	^48^
*Smittium culicis* (Zoopagomycota)	71	Yes	^49^
*Conidiobolus coronatus* (Zoopagomycota)	70	Yes	^48^
*Beauveria bassiana* (Ascomycota)	54	Yes	^48^
*Zoophthora radicans* (Zoopagomycota)	47	Yes	^50^
*Metarhizium robertsii* (Ascomycota)	39	Yes	^48^
*Zancudomyces culisetae* (Zoopagomycota)	38	Yes	^51^
*Conidiobolus thromboides* (Zoopagomycota)	37	Yes	^52^
*Metarhizium brunneum* (Ascomycota)	30	Yes	^48^
*Furculomyces boomerangus* (Zoopagomycota)	28	Yes	^51^
*Smittium simulii* (Zoopagomycota)	27	Yes	^49^
*Smittium mucronatum (*Zoopagomycota)	24	Yes	^51^
*Torrubiella hemipterigena* (Ascomycota)	22	Yes	^53^
*Linderina pennispora* (Zoopagomycota)	21	Unknown	
*Metarhizium guizhouense* (Ascomycota)	20	Yes	^54^
*Smittium angustum* (Zoopagomycota)	19	Yes	^51^
*Basidiobolus meristosporus* (Zoopagomycota)	17	Unknown	
*Smittium megazygosporum* (Zoopagomycota)	16	Yes	^55^
*Cordyceps militaris* (Ascomycota)	15	Yes	^56^

^a^Species names in this table are italicized.

## Discussion

**Fig. 5:**
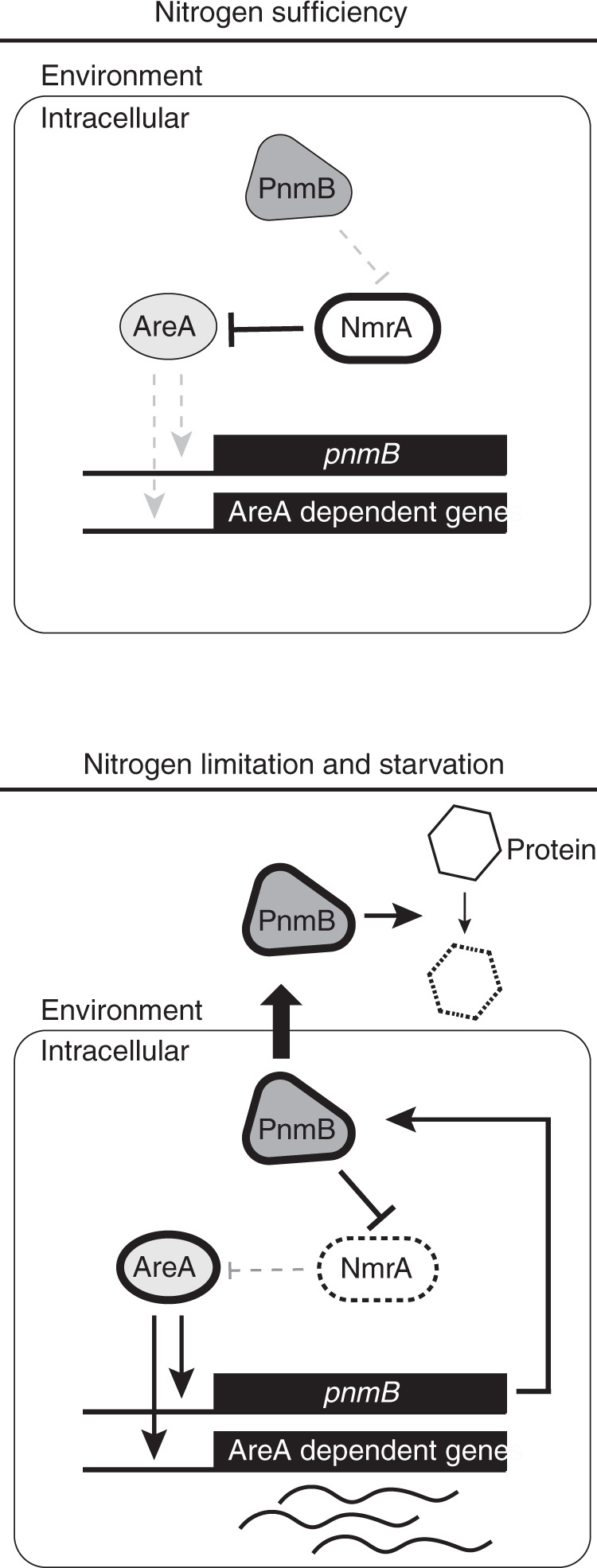
Regulatory model of AreA-NmrA-PnmB loop. In nitrogen-sufficient conditions, NmrA levels are high, while AreA levels are low. NmrA represses AreA-mediated activation of *pnmB* and nitrogen metabolism genes. In nitrogen-starvation conditions, AreA expression and activity is increased while NmrA levels are decreased. *pnmB* expression is activated by AreA. The increased PnmB levels further accelerate the decrease of NmrA levels. AreA, PnmB, and NmrA form a positive regulatory loop to enhance AreA activity and nitrogen response rate. At the same time, PnmB was also secreted outside the cell to facilitate nutrient acquisition.

This work shows that the *A. nidulans* PnmB protease has two functions in nitrogen metabolism (Fig. 5). First, it is secreted as an extracellular protease where it contributes to the degradation of proteins to facilitate nutrient breakdown and nitrogen acquisition. The second role is to degrade the co-repressor NmrA to enhance the response to nitrogen starvation by increasing the turnover of NmrA when its synthesis is reduced due to loss of activation by MeaB^19^. The dual functionality is clearly not essential in all fungi because ~20% of PnmB-like protein sequences do not carry a signal peptide for secretion (Supplementary Data 1), but presumably provides a selective advantage in some environments such as an osmotrophic ecosystem^7^ where nitrogen liberated by extracellular PnmB-mediated proteolysis may be competed for by other cohabiting microorganisms. It is tempting to speculate that those PnmB homologs without a signal peptide may also control NmrA turnover and that this is the only role in the respective fungal species. The coupling of extracellular proteolysis with intracellular NmrA degradation (and hence, relief of repression by NmrA of AreA-dependent activation of nutrient acquisition and metabolism genes) could ensure rapid nitrogen nutrient uptake in order to protect the released nutrients from competitors. It appears that the *pnmB* gene might offer a selective advantage in niches occupied by both fungi and insects or for fungi with insect hosts, based on the moderate to large expansions of this gene in many entomo-fungi.

How the protease gene evolved the two PnmB functions in *A. nidulans* is a fascinating question. As most fungal PnmB-like proteins (~80%) carry a signal peptide (Supplementary Data 1), the ancestral gene likely encodes an extracellular protease for protein breakdown, while the second transcription regulatory function was newly evolved. This new function would require evolution to establish an intracellular population of PnmB in addition to the secreted form. One possibility is that secretion of the ancestral protease may not have been very efficient and poor secretion may have led to an intracellular population. Acquisition of the transcriptional regulation role would have provided selection to maintain poor secretion. Alternatively, if secretion of the ancestral protease was efficient, mutations affecting efficient recognition of the signal peptide sequence or establishing two forms differing at the N-terminus with and without a signal peptide could lead to a switch from secretion to both secretion and intracellular retention. We observed two distinct intracellular isoforms of PnmB^HA^ protein. Our analysis suggests that they are not a result of alternative transcript isoforms. An internal methionine codon (codon 18) might act as an internal translation start site^35^ to generate the intracellular form of PnmB lacking a signal peptide.

Another notable difference between the extracellular and intracellular forms of PnmB is their substrate specificity. Our results showed that the secreted PnmB has broad-spectrum proteolytic activity able to degrade milk proteins, whereas intracellular PnmB is highly specific for NmrA with no sign of non-specific degradation of histone and total proteins. A previous in vitro study had identified a specific PnmB cleavage site on NmrA^27^. It is currently unclear how the specificity for NmrA (or the loss of broad-spectrum activity) is achieved by intracellular PnmB, but it is likely mediated by direct interaction between PnmB and NmrA.

PnmB activities are controlled at the level of its expression according to nitrogen availability (this study, ref. ^27^). The global nitrogen regulator AreA is absolutely required for *pnmB* expression through direct binding to the *pnmB* promoter. We have further demonstrated that this positive regulation by AreA in turn creates a positive feedback regulatory loop through degrading the co-repressor NmrA to accelerate AreA activation kinetics upon nitrogen starvation (Fig. 5). Consequently, genes required for nitrogen breakdown, uptake and metabolism are promptly induced such that *A. nidulans* can quickly scavenge nitrogen nutrients in the environment. Therefore, our work has identified a mechanism for eliciting a rapid transcription response for nitrogen starvation. It is noteworthy that two more unknown proteases that cleave NmrA were implicated in NmrA turnover^27^. It will be interesting to see whether they serve similar roles as PnmB. As *A. nidulans* often lives with many other microbes in the wild, the accelerated transcription response would provide a growth advantage in competitive and nutrient-limiting environments. Hence, our study reveals a protease gene that plays a role in essential physiology in the cell to enhance its growth competitiveness in nature.

## Methods

### Strains, media, and primers used in this study

*A. nidulans* strains used and their genotypes are listed in Table 2. MH11726 was obtained by outcross from MH11626^19^. RT303 was obtained by outcross of MH9641^14^. Strain constructions described below used the gene-targeting *nkuA*∆ recipient strain MH11036^36^. *Aspergillus* nitrogen-free medium (ANM) containing supplements and indicated carbon and nitrogen sources was used for liquid and solid growth experiments^37^. Sequences of primers used in this study are listed in Table 3.

**Table 2:** *A. nidulans* strains used in this study.

Strain	Genotype^a^	Origin/reference
CWF579	*pyroA4 nkuA*∆*::argB; pnmB*∆*::Bar; riboB2*	This study
CWF583	*pyroA4 nkuA*∆*::argB; pnmB*^*HA*^*-Bar; riboB2*	This study
CWF598	*yA::xylP*(p)*pnmB-AfpyroA; pyroA4 nkuA*∆*::argB; riboB2*	This study
CWF599	*yA::xylP*(p)*prtA-AfpyroA; pyroA4 nkuA*∆*::argB; riboB2*	This study
CWF611	*yA::nmrA*^*FLAG*^*-AfpyroA; nmrA*Δ*::Ble*^R^*; pyroA4 nkuA*∆*::argB; pnmB::Bar*	This study
CWF664	*yA::xylP*(p)*prtA*^*HA*^*-AfpyroA; pyroA4; nkuA*∆*::argB; riboB2*	This study
CWF666	*yA::nmrA*^*FLAG*^*-AfpyroA; nmrA*Δ*::Ble*^R^ *wA::xylP*(p)*pnmB*^*HA*^*-AfriboB; amdS-lacZ; pyroA4 nkuA*∆*::Bar; riboB2*	This study
MH5699	*yA1 adE20 su(adE20); areA*∆*::riboB; pyroA4; riboB2*	^57^
MH8935	*yA1; nmrA*∆*::Ble*^R^*; amdS::lacZ; pyroA4*	^25^
MH11036	*pyroA*4 *nkuA*∆*::argB*; *riboB2*	^36^
MH11626	*yA::nmrA*^*FLAG*^*-AfpyroA; nmrA*Δ*::Ble*^R^*; amdS-lacZ; pyroA4 nkuA*∆*::Bar; riboB2*	^19^
MH11726	*yA::nmrA*^*FLAG*^*-AfpyroA; nmrA*Δ*::Ble*^R^*; pyroA4 nkuA*∆*::Bar*	This study
RT303	*areA*^*HA*^	This study

^a^Gene names in this table are italicized.

**Table 3:** Primers used in this study.

Primer name	Primer sequence
PnmB_delF1	ATATCGAATTCCTGCAGCCCCATCGGAAATGGTGCAAAAT
PnmB_delR1	GTTTAAACGAGCTCGAATTCCTCTCGGGGAGGGGGAGCGA
PnmB_delF2	GTTTAAACGAGCTCGAATTCGCGGTTTCGTTGAGATCTAG
PnmB_delR2	CTAGAACTAGTGGATCCCCCCTGATACGTGACACCGTCAC
PnmB_tagF1	ATATCGAATTCCTGCAGCCCTTCATTCAATCCTCACGAAG
PnmB_tagR1	TTAATTAACCCGGGGATCCGCAGACCAGTAACCTGCTGGA
LongtineF	CGGATCCCCGGGTTAATTAA
LongtineR	GAATTCGAGCTCGTTTAAAC
PnmB_OE_F	ATATCGAATTCCTGCAGCCCCTCGACCGCAGAGCCGACTC
PnmB_OE_R	CTAGAACTAGTGGATCCCCCACGTGATCGCTTCGCCGGTC
PnmB_HAOE_R	TTAATTAACCCGGGGATCCGGATCTCAACGAAACCGCTTA
PrtA_OE_F	ATGCATTCGTTCAAGCGCTCATATCGAATTCCTGCAGCCC
PrtA_HAOE_R	TTAATTAACCCGGGGATCCGTTCGTCGGCACCGTTGTAGG
SmaI_left_R	GGGCTGCAGGAATTCGATAT
Bar54R	ATGTCCGCCTCGGTGGCACG
PnmB_-1303F	ATCCCATCTGCTGGTCGATC
ORFfreeF	AGCCGGGATCAATATCATCA
ORFfreeR	TGCGATGAGAAATAGCAACG
UreA_947F	CTGGACAACGGCTACTACAA
UreA_1041R	GAATAGCGAACCAGCAGAGA
MepA_124F	TAGCGTCAAGTCTGGTGATATTG
MepA_232R	TTAGTAGTGAGGGAGGGTATGG
ActA_184F	GCCAGTTCACTTCCGTGCTTGT
ActA_284R	CGAACGCCAATGCCCAACCA
BenA_391F	ACACGACACCTCAACAGAAC
BenA_516R	CACACCAAACTGCTACCTGATA
PnmB_-1116R	TCGCTGATAGAGGCCCGACC
PnmB_337F	GAGCTGGCAGAAGAGCTCCA
PnmB_509R	GTCACTCGAGCACTGGCTCT
PnmB_-1047F	GGAAGCGGAAAAGGTGCCCT
PnmB_-900R	AAGAAACGCCTCTCCACGTCG
PnmB_-259F	CACAGCCCCAGCAGTGTTGT
PnmB_-125R	CTAGGTCGGTGCGCGTGTTC
PnmB_-103F	GGAGTCAATTCTGCTCTGCAG
PnmB_6R	CTTCATTTTGACGGCGCGA
PnmB_-558F	CTACTCGATAGGCCACCCAG
PnmB_-456R	ATCCCATCTGCTGGTCGATC
PnmB_-726F	AGTGCTTACGGCCATTTCAC
PnmB_-549R	ATCGAGTAGGTAGAGACAGG

### Deletion of *pnmB*

The *pnmB* deletion strain (CWF579) was generated by replacing the entire *pnmB* coding region with the *Bar* gene from *S. hygroscopicus* that confers glufosinate resistance. The deletion construct was generated as described previously^38^ using primers PnmB_delF1, PnmB_delR1, PnmB_delF2, and PnmB_delR2 and subsequently amplified using LongtineF and LongtineR primers. The resultant DNA construct was transformed into MH11036 (*pyroA4*, *riboB2*, *nkuA*∆*::argB*), and glufosinate-resistant transformants were isolated. Deletion of *pnmB* was confirmed in one transformant (CWF579) by Southern blot analysis (Supplementary Fig. 8).

### Generation of the PnmB^HA^ strain

The DNA construct inserting a HA epitope tag at the C terminus of PnmB was generated as described previously^38^ using the primers PnmB_tagF1, PnmB_tagR1, PnmB_delF2, PnmB_delR2, LongtineF, and LongtineR. The DNA construct was transformed into MH11036, and glufosinate-resistant transformants were isolated. One transformant (CWF583) was confirmed by Southern blot analysis for correct integration (Supplementary Fig. 8) and by western blot analysis for successful tagging.

### Generation of *pnmB*^*HA*^ and *prtA*^*HA*^ overexpression strains

The coding region of *pnmB* or *prtA* was amplified by PCR using primers PnmB_OE_F, PnmB_HAOE_R, PrtA_OE_F and PrtA_HAOE_R specified in Table 3. The *pnmB* and *prtA* PCR products were introduced after the *xylP* promoter in the pCWB588 plasmids, (which also contain a 5′ and 3′ truncated *yA* gene fragment for targeting to the *yA* locus) using the Isothermal Assembly method^39^. The resultant *xylP*(p)::*pnmB*^HA^ (pCWB288) and *xylP*(p)::*prtA*^HA^ (pCWB290) constructs were verified by Sanger-sequencing and transformed into MH11036. The *xylP*(p)::*pnmB*^HA^ (CWF598) and *xylP*(p)::*prtA*^HA^ (CWF664) transformants were tested for overexpression by western blot analysis. For *pnmB*, another overexpression strain (CWF666) was created by targeting the *xylP*(p)-*pnmB*^HA^ overexpression construct (pCWB287), which contains a 5′ and 3′ truncated *wA* fragment, to the *wA* locus in the CWF387 parent strain.

### Growth tests

Strains were point-inoculated and grown on solid ANM^37^ containing supplements and the indicated nitrogen source at 10 mM (except where indicated) under the specified temperature and pH conditions and with or without 10 mM TU (Sigma) for 2 days. For quantification in the TU derepression assay, the average of three colony diameter measurements was calculated for the strains in each growth assay. Three independent assays were carried out. The ratio was calculated by dividing the average colony diameter on the xylose condition by that under the glucose condition. Statistical significance was calculated using *t* test in GraphPad of Prism 5. For phenotypic assessment of *pnmB* overexpression, 1% xylose was also added to the media to induce overexpression.

### Western blot analysis

Strains were grown in 100 mL ANM liquid medium with supplements and 10 mM ammonium tartrate at 37 °C for 16 h. For nitrogen starvation, mycelia were washed with and transferred to pre-warmed nitrogen-free ANM media for the indicated amount of time. Xylose was added to the media at a final concentration of 1% to induce PnmB and PrtA overexpression in the respective overexpression strains. Mycelia were harvested on Miracloth, pressed dried, snap-frozen in liquid nitrogen, and kept at −80 °C until protein extraction. Total proteins were extracted as previously described^19^. In all, 50 μg of total proteins were separated in 10% SDS-PAGE gel and transferred to PVDF membrane for immuno-detection. PnmB^HA^, NmrA^FLAG^, and histone H3 were detected using HA-probe (Santa Cruz sc-7392), anti-FLAG® M2 (Sigma F1804), and anti-Histone H3 (Abcam ab1791) antibodies, respectively, at the concentration of 0.1 µg/mL. Goat anti-mouse (Millipore AP124P) or goat anti-rabbit horseradish peroxidase (HRP)-conjugated (Millipore AP132P) antibodies were used as the secondary antibody at the concentration of 0.1 µg/mL for Chemiluminescence detection using Clarity™ Western ECL Substrate (Bio-rad 1705060) kit.

### Co-IP of NmrA^FLAG^ and PnmB^HA^

Strains expressing NmrA^FLAG^ (MH11626)^19^, PnmB^HA^ (CWF664), or both NmrA^FLAG^ and PnmB^HA^ (CWF666) were grown in 100 mL ANM media containing supplements and 10 mM ammonium tartrate at 37 °C for 16 h. Overexpression of PnmB^HA^ was induced with 1% xylose for 4 h at 37 °C. Mycelia were collected by filtering using a Mira-cloth, immediately frozen in liquid nitrogen and then ground into fine powder in liquid nitrogen using a mortar and pestle. Ground mycelia powder was then transferred into a fresh tube containing zirconium beads (~100 μL in volume) and subjected to 5 cycles of 3-min lysis at the maximum speed using a Bullet Blender in 1 mL IP buffer (250 mM NaCl, 100 mM Tris-HCl pH 7.5, 10% glycerol, 1 mM EDTA, and 0.1% NP-40). Protease inhibitors (1 mM PMSF, 1x Roche EDTA-free cOmplete-mini Protease Inhibitor Cocktail containing benzamidine), phosphatase inhibitors (100 mM NaF, 50 mM Na vanadate, 80 mM β-glycerol phosphate), and 1.5 mM dithiothreitol were added immediately before lysis. For IP, 1 mL of total protein lysate was incubated with 2 µg mouse anti-FLAG (Sigma F3165) or mouse anti-HA (Santa Cruz SC-7392) antibody with gentle rotation for 2 h at 4 °C. 10 µL of pre-washed protein A beads (GE Healthcare 17-0963-03) was added and the mixture was incubated for additional 2 h with gentle rotation. The protein A beads were washed 5 times with 1 mL of IP buffer. In the final round, beads were resuspended in 50 µL of 4× Laemmli protein loading buffer and incubated at 95 °C for 10 min for elution. The immuno-precipitated samples were separated on a 10% SDS-PAGE gel for western blot analysis using mouse anti-FLAG HRP-conjugated antibody (Abcam ab49763) for NmrA^FLAG^ and rabbit anti-HA (Abcam ab9110) primary antibody followed by goat anti-rabbit HRP-conjugated secondary antibody (Merck Millipore, AP132P) for PnmB^HA^ at the concentration of 0.1 µg/mL.

### Protein secretion analysis

Strains were first grown in ANM media containing supplements and 10 mM ammonium tartrate at 37 °C for 16 h, and then transferred to 10 ml nitrogen-free ANM media for an additional 4 h. For PnmB and PrtA overexpression, 1% xylose was added to the media. Mycelia were collected by filter with miracloth, and mycelia were subjected to total protein extraction as the intracellular fraction the TCA extraction method described above, while the conditioned growth media was further filtered using a 0.2 μm filter to remove all mycelia before protein extraction by a modified TCA method. Briefly, 10 μg BSA and 1 mL of ice-cold 100% TCA was added to 5 mL of filtered medium, and then 2 ml of the mixture was transferred to a microfuge tube and centrifuged at 4 °C 15000 rpm for 30 min. The protein pellet was washed with 1 mL ice-cold acetone and air-dried, followed by re-suspension in 50 μL protein loading buffer with incubation at 95 °C for 10 min. 50 μL secreted protein and 10 μg intracellular total protein was separated in 10% SDS-PAGE and subjected to western blot analysis using HA-probe (Santa Cruz sc-7392) and anti-Histone H3 (Abcam ab1791) antibodies at the concentration of 0.1 µg/mL.

### ChIP and ChIP-seq analysis

Strains were cultured as described in the “Western blot analysis” section. Crosslinking and chromatin preparation were carried out according to a previous study^10^. Briefly, formaldehyde was added to the growth media at a final concentration of 1%, followed by gentle shaking for 20 min at room temperature. Subsequently, 25 mL 2.5 M glycine was added to terminate crosslinking and the mixture was left at room temperature for 10 min with gentle shaking. Mycelia were washed with ice-cold water and harvest by filtering on Miracloth. Harvested mycelia were press-dried, snap-frozen in liquid nitrogen, and then lyophilized for 1–2 h before storing at −80 °C for chromatin preparation. Lyophilized mycelia were lysed with zirconium beads in ice-cold FA lysis buffer for five times of 3 min in a Bullet Blender. Extracts were recovered and centrifuged to remove the supernatant. Pellets were re-suspended in cold FA lysis buffer and subjected to sonication at 100% amplitude with 10 s on and off cycles for a total sonication time of 20 min using the Qsonica Q800R machine. For IP, 2 µg of anti-RNA polymerase II antibody (Millipore 04-1572) was used for ChIP as described previously^40^. Quantitative real-time PCR was performed using primers listed in Table 3. IP efficiency was calculated for each gene by comparing the amount between IP product and input DNA. Open reading frame-free region was used as background control to compare the IP efficiency between different genes and samples. Library preparation for PolII ChIP-seq analysis was carried out as described previously^41^. Sequencing was performed on Illumina HiSeq2500. Raw reads were mapped to *A. nidulans* genome version *A_nidulans*_FGSC_A4_version_s10-m04-r03 using Bowtie2^42,43^. PolII ChIPseq signals were calculated by first summing the total number of reads overlapping at each base pair across the coding region of genes and then normalizing to gene length and total number of mapped reads number. The Integrated Genome Browser program was used to visualize the data^44^.

### Protein sequence and BLASTP analysis

PnmB motifs information was acquired from AspGD^42^. Signal peptide prediction was performed via SignalP5.0^32^. BLASTP analysis of PnmB was performed at NCBI^45^ against the non-redundant Protein Sequence (fungi) database with default parameters except for the maximum target sequences set at 5,000 and at the JGI MycoCosm database^46^. The BLASTP output hits from NCBI and JGI were combined and assigned to the 6 phyla shown in Fig. 4 and Supplementary Data 1. Taxonomy information of BLAST hits was assigned using the taxonomy database from NCBI (https://www.ncbi.nlm.nih.gov/taxonomy).

### Statistics and reproducibility

Statistics of colony diameter measurements was performed by one-tailed unpaired *t* test using the GraphPad Prism 5 software.

### Reporting summary

Further information on research design is available in the Nature Research Reporting Summary linked to this article.

## Supplementary information

Supplementary Information

Description of Additional Supplementary Files

Supplementary Data 1

Supplementary Data 2

Supplementary Data 3

Reporting Summary

## Data Availability

The PolII ChIPseq data are available from NCBI SRA database under the accession number PRJNA560791. The full blot images for all western blot analyses are included in Supplementary Fig. 9. Source data for graphs and charts can be found in Supplementary Data 3.
