# Supplementary material for: Co-option of an extracellular protease for transcriptional control of nutrient degradation in the fungus Aspergillus nidulans
Source: Commun Biol. 2021 Dec 17;4:1409. doi: 10.1038/s42003-021-02925-1 (PMC8683493; doi:10.1038/s42003-021-02925-1)
Supplement: Supplementary file 1 — Supplementary Information [file 42003_2021_2925_MOESM1_ESM.pdf]

Supplementary Fig. 1

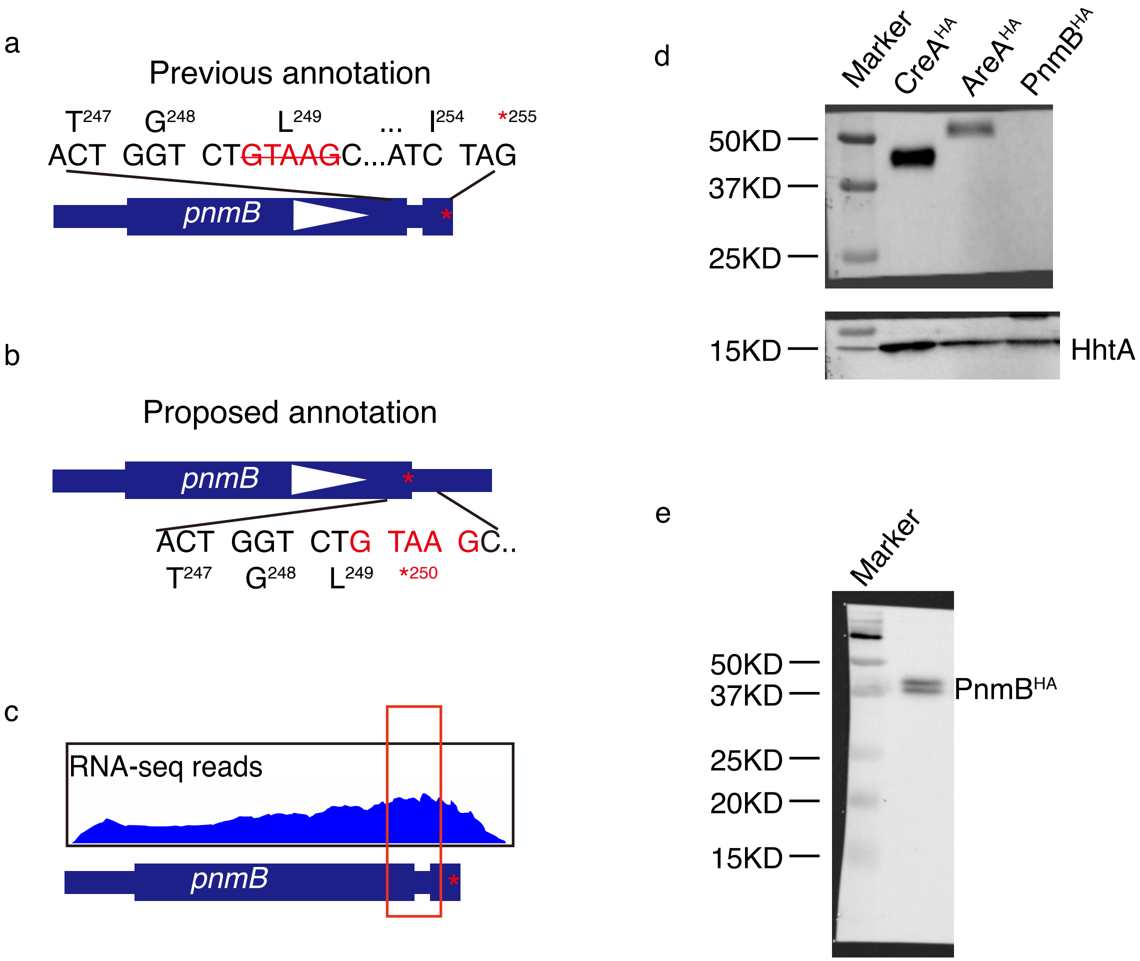

Supplementary Fig. 1. Re-annotation of *pnmB*. (a) Annotation of *pnmB* from AspGD shows a 5 bp intron highlighted in red. (b) Revised annotation of *pnmB*. (c) A genome browser screenshot of a published RNA-seq data over the *pnmB* gene. (d) Western Blot result of C-terminal tagging of PnmB based on the previous *pnmB* anno-tation. CreA and AreA are used as positive control and histone H3 protein (HhtA) is used as loading control. (e) Western Blot result of C-terminal tagging of PnmB based on the proposed *pnmB* annotation.

Supplementary Fig. 2

1 MKTAVKTTALLSLLSTAMADKAIVGGDDAEITEYPYQIALLSGGSLICGGSIISSKYVVT
61 AGHCTDGASASSLSIRAGSTYHDKGGTVVDVEAITVHPEYNANTVDNDISILELAEELQF
121 GDGIKAIDLPSSSSLPSEGTIGTATGWGALTEGGNVSPNLQYVEVPVVSKSQCSSDYSGF
181 NEITASMFCAGEEEGGKDGCQGDSGGPFAADGVLIGITSWGNGCARAGYPGVYSSPAYFR
241 DFIQQVTGL

Supplementary Fig. 2. Re-annotated PnmB protein sequence. *pnmB* encodes a protein containing 249 amino acids.

Supplementary Fig. 3

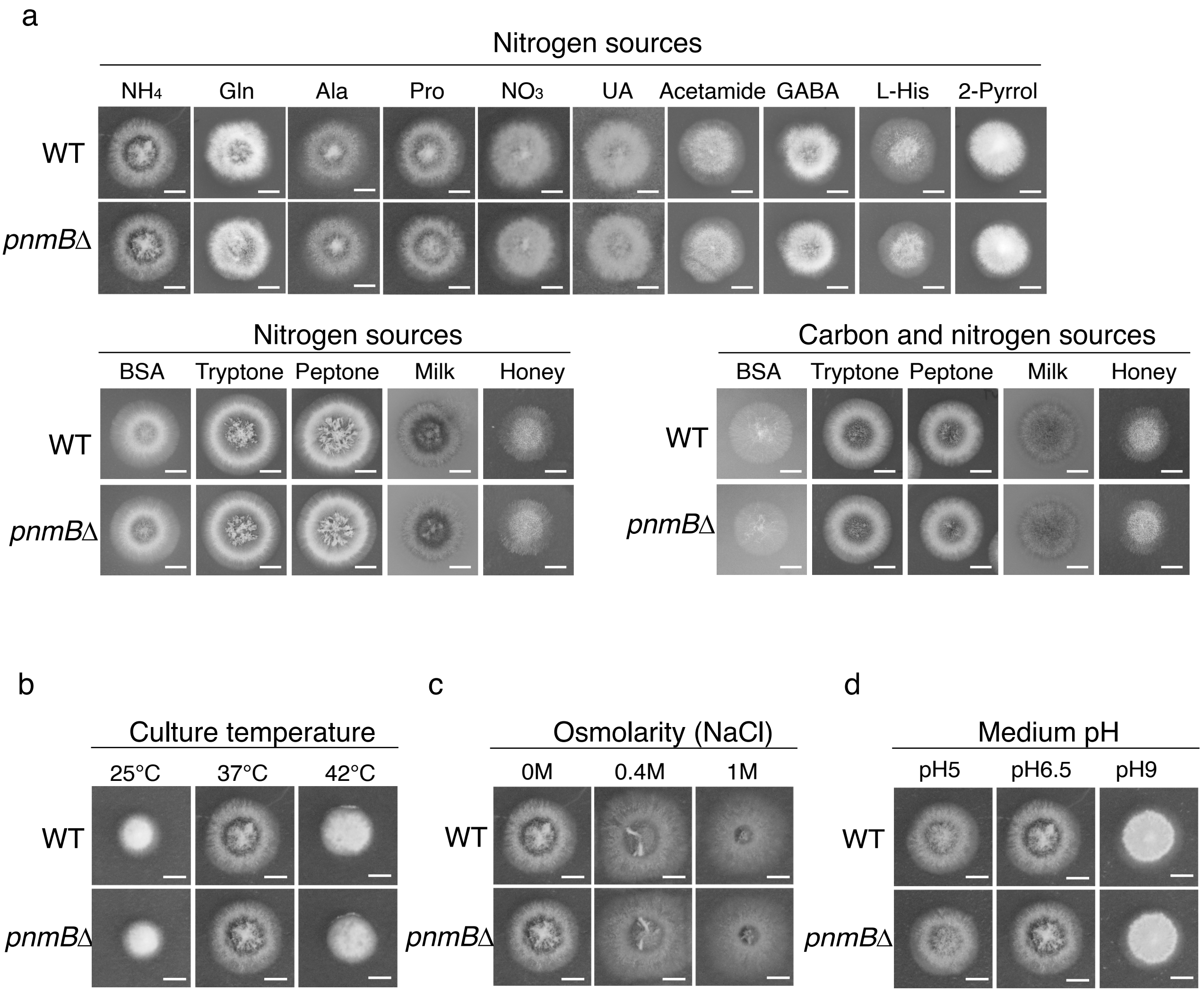

Supplementary Fig. 3. Phenotypic analysis of *pnmB* Δ. (a) different nitrogen sources (NH_4: 10 mM ammonium tartrate; Ala: 10 mM Alanine; Pro: 10mM Proline; NO_3: 10 mM Sodium nitrate; UA: 10 mM Uric acid; Gln: 10 mM glutamine; Acetamide: 10 mM Acetamide; GABA: 10 mM γ-Aminobutyric acid; L-His: 5 mM L-Histidine; 2-Pyrrol: 10 mM 2-Pyrrolidinone; BSA: 1% Bovine Serum Albumin; Tryptone: 1% Tryptone; Peptone: 1% Peptone; Milk: 1% Skim milk powder; Honey: 25% Honey), (b) different temperature (25˚C, 37˚C and 42˚C), (c) different levels of osmotic stress (0.4 M and 1 M NaCl) and (d) different pH (pH 5.0 and pH 9.0). Strains were grown for two days at 37˚C in the presence of 10 mM ammonium unless stated otherwise. The scale bar showed in each colony represents 5 mm.

Supplementary Fig. 4

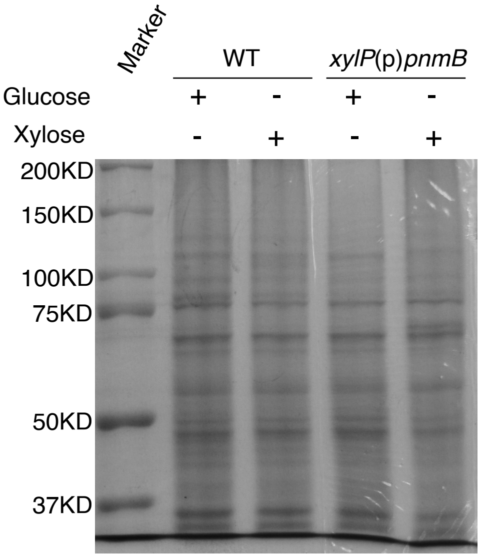

Supplementary Fig. 4. Over-expression of PnmB does not non-specifically degrade total proteins but results in a derepression phenotype. Coomassie Blue stained SDS-PAGE gel resolving total protein extracts of WT and *xylP*(p)*pnmB* strain under glucose and xylose condition.

Supplementary Fig. 5

a

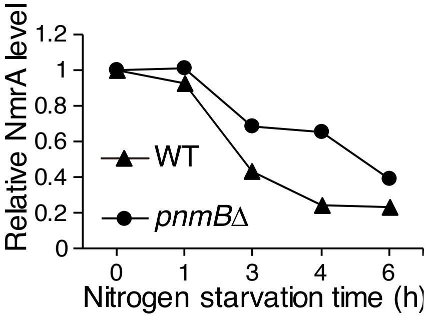

b

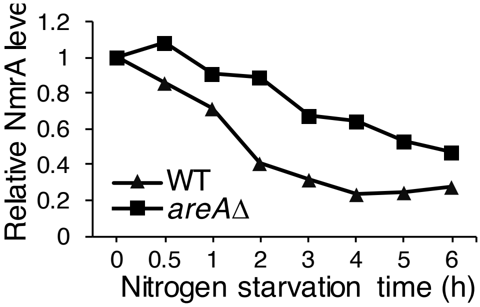

Supplementary Fig. 5. Quantification of Western Blot results. Quantification of NmrA-FLAG in (a) Figure 1c and (b) Figure 2d by densitometry comparisons of Western bands.

Supplementary Fig. 6

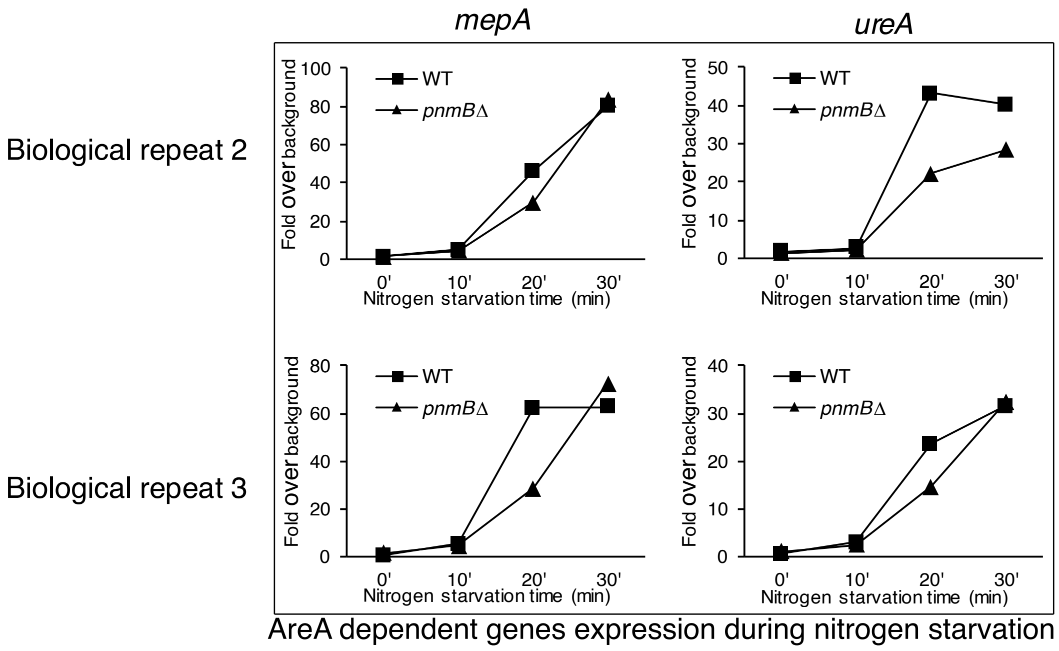

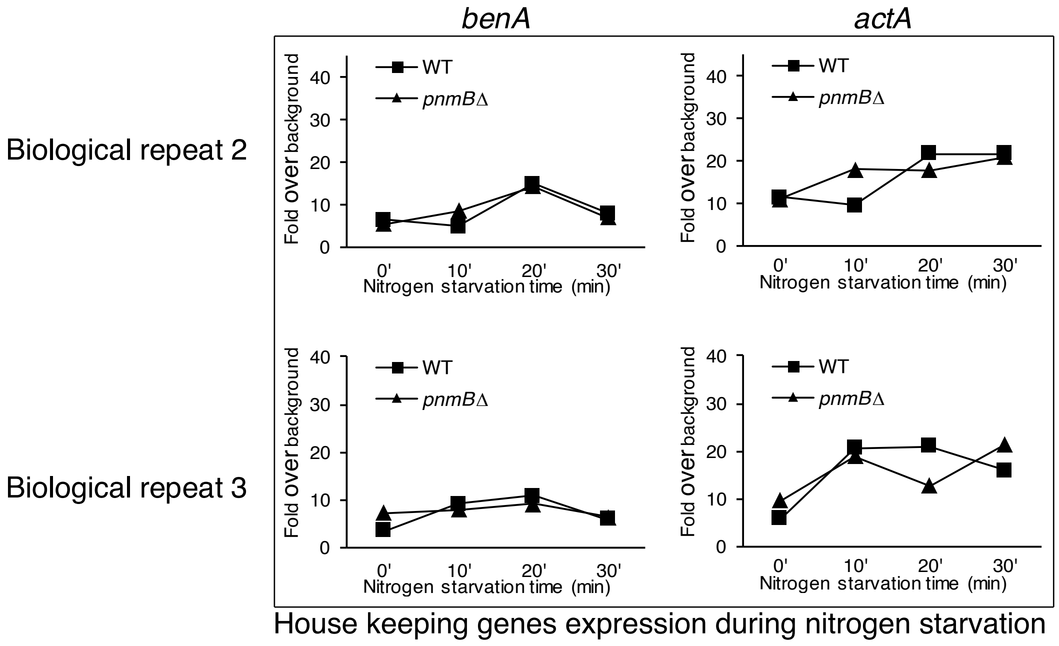

Supplementary Fig. 6. Biological repeats of PolII ChIP-qPCR analysis to measure AreA activation during nitrogen starvation. Transcription level of AreA-regulated (*mepA* and *ureA*) and house-keeping (*benA* and *actA*) genes was measured in WT and *pnmB*Δ strains during nitrogen starvation by PolII ChIP-qPCR analysis in three independent experiments. Biological repeat 2 and 3 are shown here. PolII bindings are expressed as fold over background.

Supplementary Fig. 7

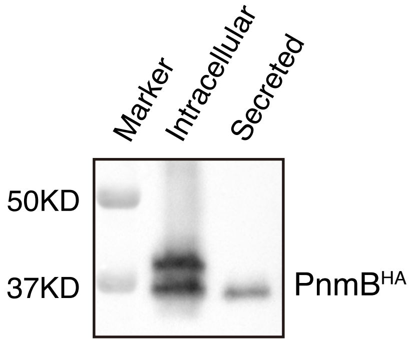

Supplementary Fig. 7. Western blot analysis of intracellular and secreted $PnmB^{HA}$. Western Blot analysis of $PnmB^{HA}$ from the intracellular and secreted fractions with extended electrophoresis time to compare the mobility of secreted and intracellular PnmB.

Supplementary Fig. 8

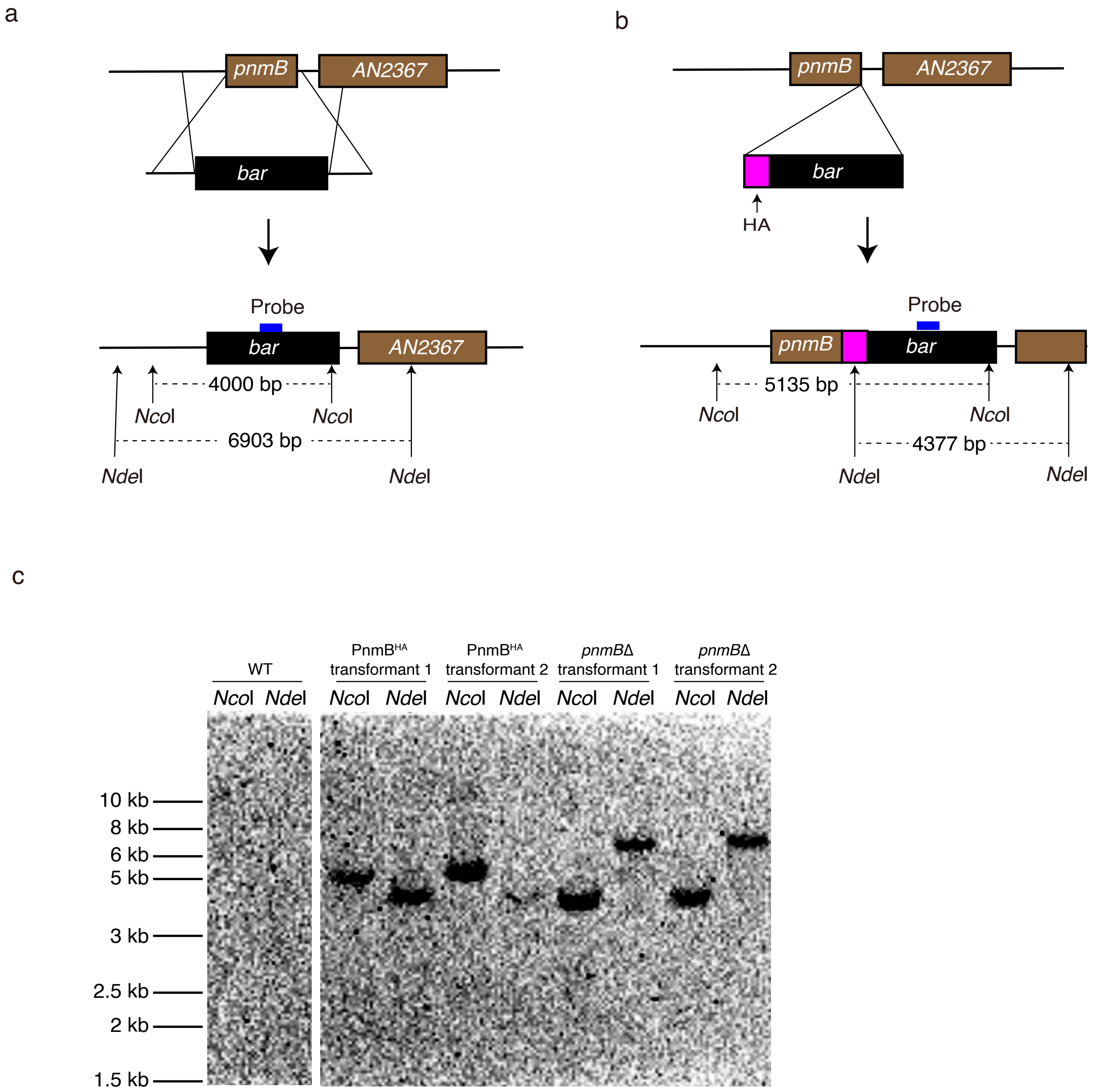

Supplementary Fig. 8. Deletion and epitope tagging of *pnmB*. (a) A schematic diagram showing *pnmB* deletion through homologous recombination. The coding region of *pnmB* is replaced by the glufosinate resistant *bar* gene. The blue box marks the probe region used in southern blot analysis. The restriction sites of *Nco*I and *Nde*I are marked by black arrows. (b) A schematic diagram showing the generation of the $PnmB^{HA}$ strain. The sequence encoding the HA epitope tag and the *bar* gene are introduced before the stop codon of the *pnmB* gene. The blue box marked the probe region used for southern blot analysis. The restriction sites of *Nco*I and *Nde*I are indicated by black arrows. (c) Southern blot of wildtype and glufosinate resistant transformants of *pnmB* deletion and tagging. All samples were run on the same gel. The original image is shown in Supplementary Data 3.

Supplementary Fig. 9

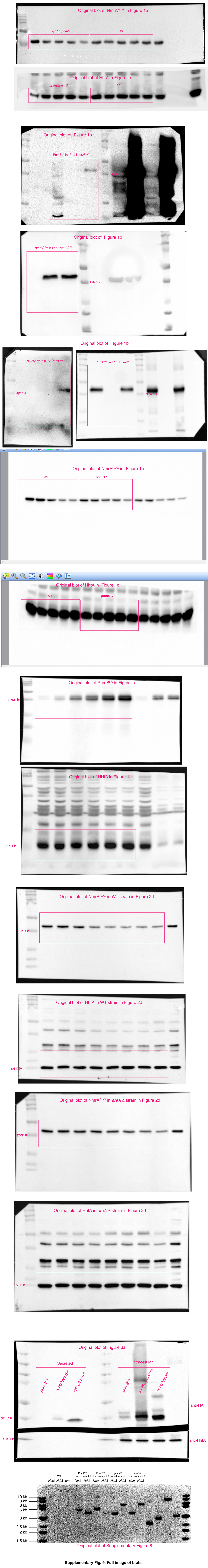

Supplementary Fig. 9. Full image of blots.
