# Supplementary material for: Co-option of an extracellular protease for transcriptional control of nutrient degradation in the fungus Aspergillus nidulans
Source: Commun Biol. 2021 Dec 17;4:1409. doi: 10.1038/s42003-021-02925-1 (PMC8683493; doi:10.1038/s42003-021-02925-1)
Supplement: Supplementary file 2 — Description of Additional Supplementary Files [file 42003_2021_2925_MOESM2_ESM.pdf]

Description of Additional Supplementary Files

File name: Supplementary Data 1

Description: PnmB-like proteins identified by BLASTP.

File name: Supplementary Data 2

Description: PnmB-like protein number in each species identified by BLASTP.

File name: Supplementary Data 3

Description: Source data for graphs and charts.
