# Supplementary material for: Co-option of an extracellular protease for transcriptional control of nutrient degradation in the fungus Aspergillus nidulans
Source: Commun Biol. 2021 Dec 17;4:1409. doi: 10.1038/s42003-021-02925-1 (PMC8683493; doi:10.1038/s42003-021-02925-1)
Supplement: Supplementary file 6 — Reporting Summary [file 42003_2021_2925_MOESM6_ESM.pdf]

nature research

Corresponding author(s): Koon Ho Wong

Last updated by author(s): Oct 4, 2021

Reporting Summary

Nature Research wishes to improve the reproducibility of the work that we publish. This form provides structure for consistency and transparency in reporting. For further information on Nature Research policies, see Authors & Referees and the Editorial Policy Checklist .

Statistics

For all statistical analyses, confirm that the following items are present in the figure legend, table legend, main text, or Methods section.

n/a	Confirmed	
☐	☒	The exact sample size (*n*) for each experimental group/condition, given as a discrete number and unit of measurement
☐	☒	A statement on whether measurements were taken from distinct samples or whether the same sample was measured repeatedly
☐	☒	The statistical test(s) used AND whether they are one- or two-sided<br>*Only common tests should be described solely by name; describe more complex techniques in the Methods section.*
☒	☐	A description of all covariates tested
☒	☐	A description of any assumptions or corrections, such as tests of normality and adjustment for multiple comparisons
☒	☐	A full description of the statistical parameters including central tendency (e.g. means) or other basic estimates (e.g. regression coefficient) AND variation (e.g. standard deviation) or associated estimates of uncertainty (e.g. confidence intervals)
☒	☐	For null hypothesis testing, the test statistic (e.g. *F*, *t*, *r*) with confidence intervals, effect sizes, degrees of freedom and *P* value noted<br>*Give P values as exact values whenever suitable.*
☒	☐	For Bayesian analysis, information on the choice of priors and Markov chain Monte Carlo settings
☒	☐	For hierarchical and complex designs, identification of the appropriate level for tests and full reporting of outcomes
☒	☐	Estimates of effect sizes (e.g. Cohen's *d*, Pearson's *r*), indicating how they were calculated

Our web collection on statistics for biologists contains articles on many of the points above.

Software and code

Policy information about availability of computer code

Data collection	No software was used to collect the data.
Data analysis	For RNA polymerase II ChIPseq analysis, the following public softwares were used:<br>FastQC was used for assessing Illumina raw read quality.<br>Bowtie2 was used for mapping raw reads to Aspergillus nidulans reference genome.<br>Integrated Genome Browser (IGB) was used for data visualization.

For manuscripts utilizing custom algorithms or software that are central to the research but not yet described in published literature, software must be made available to editors/reviewers. We strongly encourage code deposition in a community repository (e.g. GitHub). See the Nature Research guidelines for submitting code & software for further information.

Data

Policy information about availability of data

All manuscripts must include a data availability statement. This statement should provide the following information, where applicable:

- Accession codes, unique identifiers, or web links for publicly available datasets
- A list of figures that have associated raw data
- A description of any restrictions on data availability

The Pol II ChIPseq data are available from NCBI SRA database under the accession number PRJNA560791.

Field-specific reporting

Please select the one below that is the best fit for your research. If you are not sure, read the appropriate sections before making your selection.

Life sciences study design

All studies must disclose on these points even when the disclosure is negative.

Sample size	No sample size calculation was performed, because fungal strains of specific genotypes were used in the work.
Data exclusions	No data was excluded from any analysis.
Replication	All experiments had been repeated independently as biological repeats and the results are reproducible. All attempts at replication were successful.
Randomization	This is not relevant to our study. Fungal spores of indicated strains were inoculated to define culture conditions.
Blinding	Blinding was not relevant to this study, as it involves specific fungal strains and defined culture conditions.

Reporting for specific materials, systems and methods

We require information from authors about some types of materials, experimental systems and methods used in many studies. Here, indicate whether each material, system or method listed is relevant to your study. If you are not sure if a list item applies to your research, read the appropriate section before selecting a response.

Materials & experimental systems

n/a	Involved in the study	
☐	☒	Antibodies
☒	☐	Eukaryotic cell lines
☒	☐	Palaeontology
☐	☒	Animals and other organisms
☒	☐	Human research participants
☒	☐	Clinical data

Methods

n/a	Involved in the study	
☐	☒	ChIP-seq
☒	☐	Flow cytometry
☒	☐	MRI-based neuroimaging

Antibodies

Antibodies used

Santa Cruz Biotechnology, HA-probe Antibody (F-7), Cat. no. sc-7392;
Sigma, Monoclonal ANTI-FLAG® M2 antibody produced in mouse, Cat. no. F3165;
Abcam, Anti-Histone H3 antibody - Nuclear Loading Control and ChIP Grade, Cat. no. ab1791;
Merck, Anti-RNA polymerase II subunit B1 (phospho-CTD Ser-5) Antibody, clone 3E8, Cat. no. 04-1572.

Validation

Santa Cruz anti-HA:
HA-probe Antibody (F-7) is recommended for detection of proteins containing the HA tag by WB, IP, IF, FCM and ELISA.

Sigma anti-FLAG:
Antibody is recommended for use in immunoblotting, immunoprecipitation, immunocytochemistry, immunofluorescence, ELISA, electron microscopy, flow cytometry and supershift assays.

Abcam anti-Histone H3:
Suitable for: IHC-Fr, CHIPseq, Dot blot, Flow Cyt, IHC-P, Electron Microscopy, ICC/IF, ChIP, IP, WB, ChIP/Chip, IHC - Wholemount, ICC
Reacts with: Mouse, Rat, Chicken, Dog, Human, Saccharomyces cerevisiae, Xenopus laevis, Arabidopsis thaliana, Caenorhabditis elegans, Drosophila melanogaster, Ferret, Indian muntjac, Schizosaccharomyces pombe, Zebrafish, Silk worm, Dictyostelium discoideum, Rainbow trout, Trypanosoma cruzi, Neurospora crassa, Toxoplasma gondii, Rice, Schistosoma mansoni, Candida albicans, Cyanidioschyzon merolae
Predicted to work with: a wide range of other species, Mammals

Merck anti-RNA polymerase II:
Use Anti-RNA polymerase II subunit B1 (phospho-CTD Ser-5) Antibody, clone 3E8 (Rat Monoclonal Antibody) validated in WB, ELISA, ChIP to detect RNA polymerase II subunit B1 (phospho-CTD Ser-5).
Chromatin Immunoprecipitation Analysis: A representative lot was used by an independent laboratory in ChIP. (Chapman, R., et al. (2007). Science. 318(5857):1780 -1782.)
Specificity: This antibody recognizes RNA polymerase II subunit B1 at the CTD when phosphorylated at Ser5.
This antibody is widely used in studies of diverse organisms.

Animals and other organisms

Policy information about studies involving animals; ARRIVE guidelines recommended for reporting animal research

Laboratory animals	The study did not involve laboratory animals
Wild animals	The study did not involve wild animals
Field-collected samples	The study did not involve samples collected from the field
Ethics oversight	No ethical approval was required, because the work only uses a laboratory model fungus strain of Aspergillus nidulans.

Note that full information on the approval of the study protocol must also be provided in the manuscript.

ChIP-seq

Data deposition

- [ ] Confirm that both raw and final processed data have been deposited in a public database such as GEO.
- [ ] Confirm that you have deposited or provided access to graph files (e.g. BED files) for the called peaks.

Data access links<br>*May remain private before publication.*	Raw data can be found in the NCBI SRA database under the accession number PRJNA560791. Processed data and BED files can be provided upon request. For information, the RNA polymerase II ChIPseq data was used to report transcription levels of only the pnmB gene in this manuscript.
Files in database submission	WT_NH4_3E8_ATCACG_Sample_CW309-CW359.R1.fastq<br>WT_NH4_3E8_2ndrepeat_AACCGTGT_Sample_CW309-CW359.R1.fastq<br>WT_Ala4h_3E8_TTAGGC_Sample_CW309-CW359.R1.fastq<br>WT_Ala4h_3E8_2ndrepeat_CTCTGTCT_Sample_CW309-CW359.R1.fastq<br>WT_3E8_NF_CTCTGTCT_CW466_525.R1.fastq<br>WT_NF_3E8_2ndrepeat_CCATACAC_Sample_CW309-CW359.R1.fastq<br>areAdel_NH4_3E8_ACTTGA_Sample_CW309-CW359.R1.fastq<br>areAdel_NH4_3E8_2ndrepeat_GCAGCCTC_Sample_CW309-CW359.R1.fastq<br>areAdel_Ala4h_3E8_GATCAG_Sample_CW309-CW359.R1.fastq<br>areAdel_Ala4h_3E8_2ndrepeat_TCGCGTAC_Sample_CW309-CW359.R1.fastq<br>areAdel_NF_3E8_TAGCTT_Sample_CW309-CW359.R1.fastq<br>areAdel_NF_3E8_2ndrepeat_TATACCGT_Sample_CW309-CW359.R1.fastq
Genome browser session<br>(e.g. UCSC)	*Provide a link to an anonymized genome browser session for "Initial submission" and "Revised version" documents only, to enable peer review. Write "no longer applicable" for "Final submission" documents.*

Methodology

Replicates	Two independent biological replicates was performed for each experiment and condition. Median correlation of each pair is around 0.945. The replicates were checked for agreement by manually inspecting the data on the IGB genome browser for the gene-of-interest (pnmB, which is the only gene that uses the RNA polymerase II ChIPseq data) as well as on many house keeping genes and known target genes with positive RNA polymerase binding signals.
Sequencing depth	The median raw and mapped read numbers are 1.95 and 1.64 million, respectively, for the samples.<br>Read length is 51 base pair after trimming.<br>Single end sequencing was used.<br>The average sequencing depth at the genomic level (~30 Mb genome) is around 3x. Under the analyzed conditions, less than 10% of genes have significant RNA polymerase II binding signals. With the total number of annotated genes of 10,722 and with an average gene length of ~2kb, the sequencing depth for the RNA polymerase II bound regions is higher than that calculated for genomic coverage.
Antibodies	Merck, Anti-RNA polymerase II subunit B1 (phospho-CTD Ser-5) Antibody, clone 3E8, Cat. no. 04-1572.
Peak calling parameters	The experiment is RNA polymerase II ChIPseq, which did not involve peak calling. Binding levels for only one gene (pnmB) under different conditions was reported in this work; the number of reads mapped to the pnmB coding region was counted and normalized to total number of mapped reads.
Data quality	The experiment is RNA polymerase II ChIPseq, which involve no peak calling. Correlation and manual inspection on genome browser was used to assess data quality. In particular, we checked that the one gene (pnmB) reported in this work showing consistent results between biological replicates.
Software	The Integrated Genome Browser (IGB) program was used to visualize the data.
